# Supplementary material for: Population Genomics of Sub-Saharan Drosophila melanogaster: African Diversity and Non-African Admixture
Source: PLoS Genet. 2012 Dec 20;8(12):e1003080. doi: 10.1371/journal.pgen.1003080 (PMC3527209; doi:10.1371/journal.pgen.1003080)
Supplement: Figure S1 — Evaluation of the tradeoff between genomic coverage and error rate (estimated Phred score) for a series of nominal quality score thresholds. Resequenced genomes from the reference strain (y1 cn1 bw1 sp1) were modified to simulate realistic levels of variation. Assembly and filtering were conducted as described for the other genomes. Based on the above relationship, we chose a nominal quality score of Q31 (marked in red) to jointly maximize genomic coverage and estimated true quality score. (PDF) [file pgen.1003080.s001.pdf]

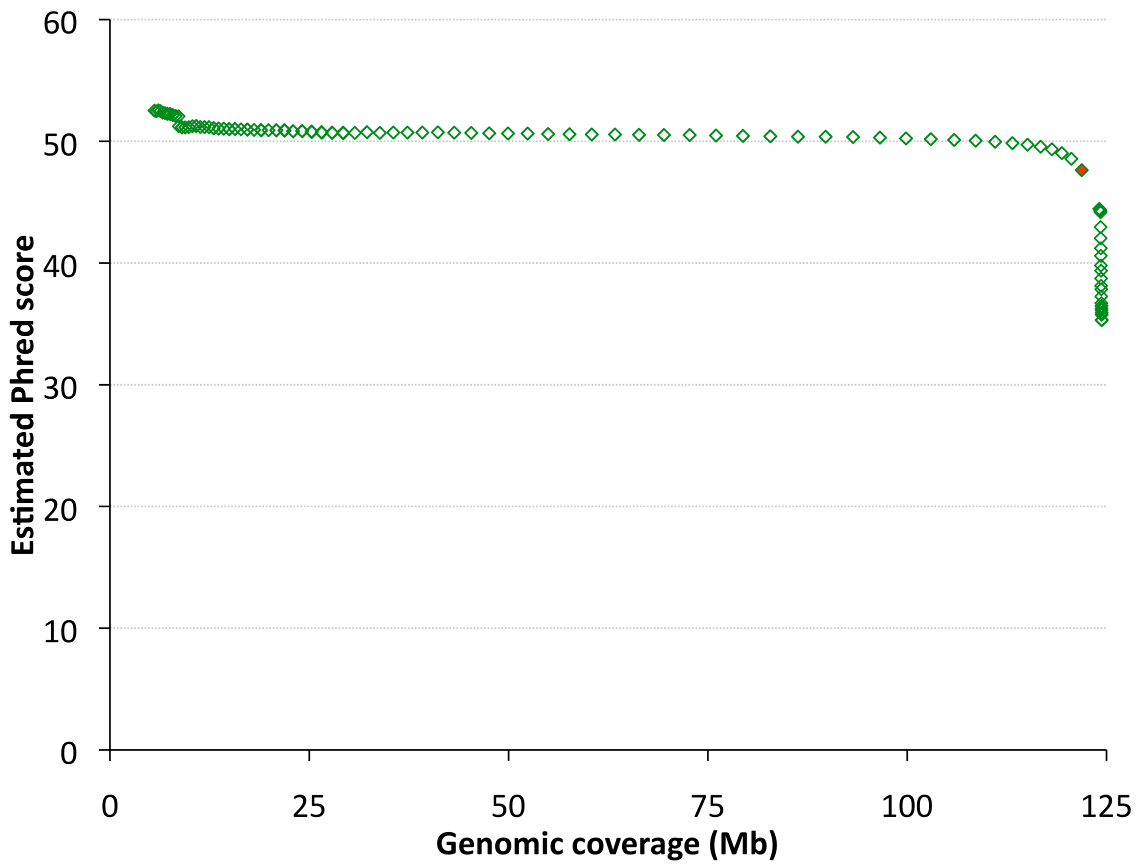

Figure S1. Evaluation of the tradeoff between genomic coverage and error rate (estimated Phred score) for a series of nominal quality score thresholds. Resequenced genomes from the reference strain (*y*[1] *cn*[1] *bw*[1] *sp*[1]) were modified to simulate realistic levels of variation. Assembly and filtering were conducted as described for the other genomes. Based on the above relationship, we chose a nominal quality score of Q31 (marked in red) to jointly maximize genomic coverage and estimated true quality score.
