# Supplementary material for: Population Genomics of Sub-Saharan Drosophila melanogaster: African Diversity and Non-African Admixture
Source: PLoS Genet. 2012 Dec 20;8(12):e1003080. doi: 10.1371/journal.pgen.1003080 (PMC3527209; doi:10.1371/journal.pgen.1003080)
Supplement: Figure S2 — A: Within-population genetic distances for 27 RG genomes, with each series representing a different sample coverage threshold. Cov2 is the absence of any threshold. Cov26 and Cov27 require that a site have a called allele (at nominal Q31) in at least 26 or all 27 of the RG genomes, respectively. Cov117 and Cov118 require that a site have a called allele in at least 117 or all 118 core genomes from all populations. Sample coverage thresholds were associated with large decreases in variation, as they preferentially excluded variable sites. The most stringent thresholds (e.g. Cov118) lessened the dependence of genetic distances on sequencing depth. B: For the 27 RG genomes, a comparison of within-population genetic distances and distance to the published reference genome. For the unfiltered data (Cov2), within-population and reference divergences are of similar magnitude for genomes with >25X depth (here, outliers for low reference divergence may represent non-African admixture). A consistent “reference bias” (closer relationship to the reference genome than to genomes from the same population) was observed for genomes with <25X depth. For the stringent sample coverage threshold (Cov118), all genomes show strong reference sequence bias. In fact, the reference sequence becomes the closest relative of each African genome. No sample coverage thresholds were used in downstream analyses. (PDF) [file pgen.1003080.s002.pdf]

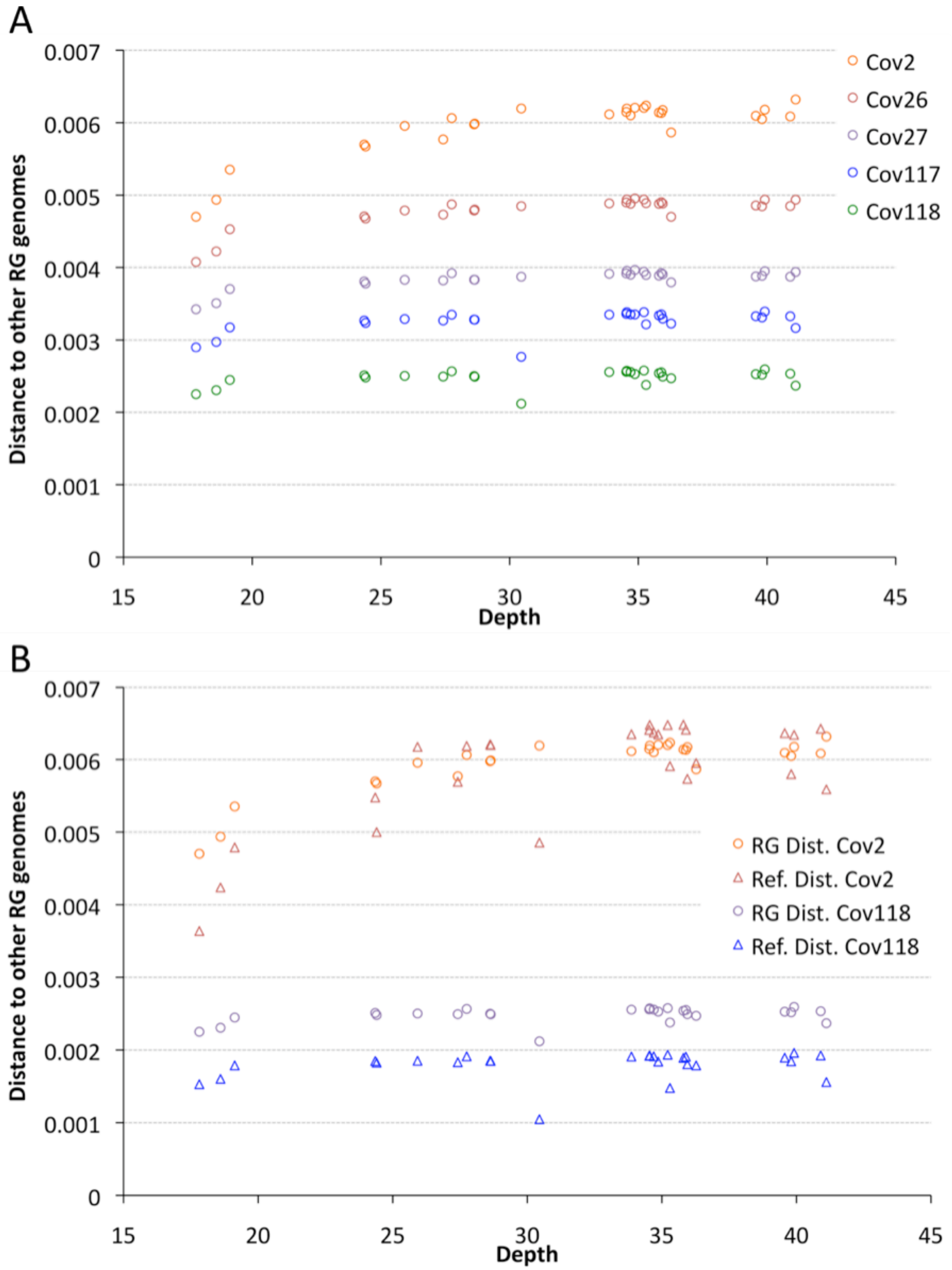
A
0.007
0.006
0.005
0.004
0.003
0.002
0.001
0
Distance to other RG genomes
15
20
25
30
35
40
45
Depth
Cov2
Cov26
Cov27
Cov117
Cov118
B
0.007
0.006
0.005
0.004
0.003
0.002
0.001
0
Distance to other RG genomes
15
20
25
30
35
40
45
Depth
RG Dist. Cov2
Ref. Dist. Cov2
RG Dist. Cov118
Ref. Dist. Cov118

Figure S2. A: Within-population genetic distances for 27 RG genomes, with each series representing a different sample coverage threshold. Cov2 is the absence of any threshold. Cov26 and Cov27 require that a site have a called allele (at nominal Q31) in at least 26 or all 27 of the RG genomes, respectively. Cov117 and Cov118 require that a site have a called allele in at least 117 or all 118 core genomes from all populations. Sample coverage thresholds were associated with large decreases in variation, as they preferentially excluded variable sites. The most stringent thresholds (e.g. Cov118) lessened the dependence of genetic distances on sequencing depth. B: For the 27 RG genomes, a comparison of within-population genetic distances and distance to the published reference genome. For the unfiltered data (Cov2), within-population and reference divergences are of similar magnitude for genomes with >25X depth (here, outliers for low reference divergence may represent non-African admixture). A consistent "reference bias" (closer relationship to the reference genome than to genomes from the same population) was observed for genomes with <25X depth. For the stringent sample coverage threshold (Cov118), all genomes show strong reference sequence bias. In fact, the reference sequence becomes the closest relative of each African genome. No sample coverage thresholds were used in downstream analyses.
