# Supplementary material for: Population Genomics of Sub-Saharan Drosophila melanogaster: African Diversity and Non-African Admixture
Source: PLoS Genet. 2012 Dec 20;8(12):e1003080. doi: 10.1371/journal.pgen.1003080 (PMC3527209; doi:10.1371/journal.pgen.1003080)
Supplement: Figure S3 — Expectations and observations for genetic distances with regard to population ancestry. (A) An illustration of basic diversity relationships between sub-Saharan and cosmopolitan populations. Cosmopolitan genetic variation is essentially a subset of that observed in sub-Saharan Africa. Due to the diversity loss associated with the out-of-Africa expansion, genetic distances amongst cosmopolitan haplotypes are lower than if cosmopolitan and sub-Saharan haplotypes are compared. The admixture inference method compares sub-Saharan and cosmopolitan genomes, assessing whether each genomic window truly looks like a “S∶C” comparison above (in the case of African ancestry for the sub-Saharan genome) or if it instead resembles a “C∶C” comparison between cosmopolitan genomes, based on genetic distance to France being lower than expected for a truly African haplotype. (B) Plots of the local ratio of DFR (genetic distance to the France sample) for single Rwanda RG genomes relative to the FR average (genetic distance among France genomes). Shown are RG2 in light green (for which no admixture was called) and RG21 in red (for which two admixture intervals were called, see yellow boxes), for windows along the complete arm 2R. As shown here, the two RG lines have generally similar genetic distances to the France sample, but within the putative admixture intervals, RG21 becomes more similar to the cosmopolitan genomes. (PDF) [file pgen.1003080.s003.pdf]

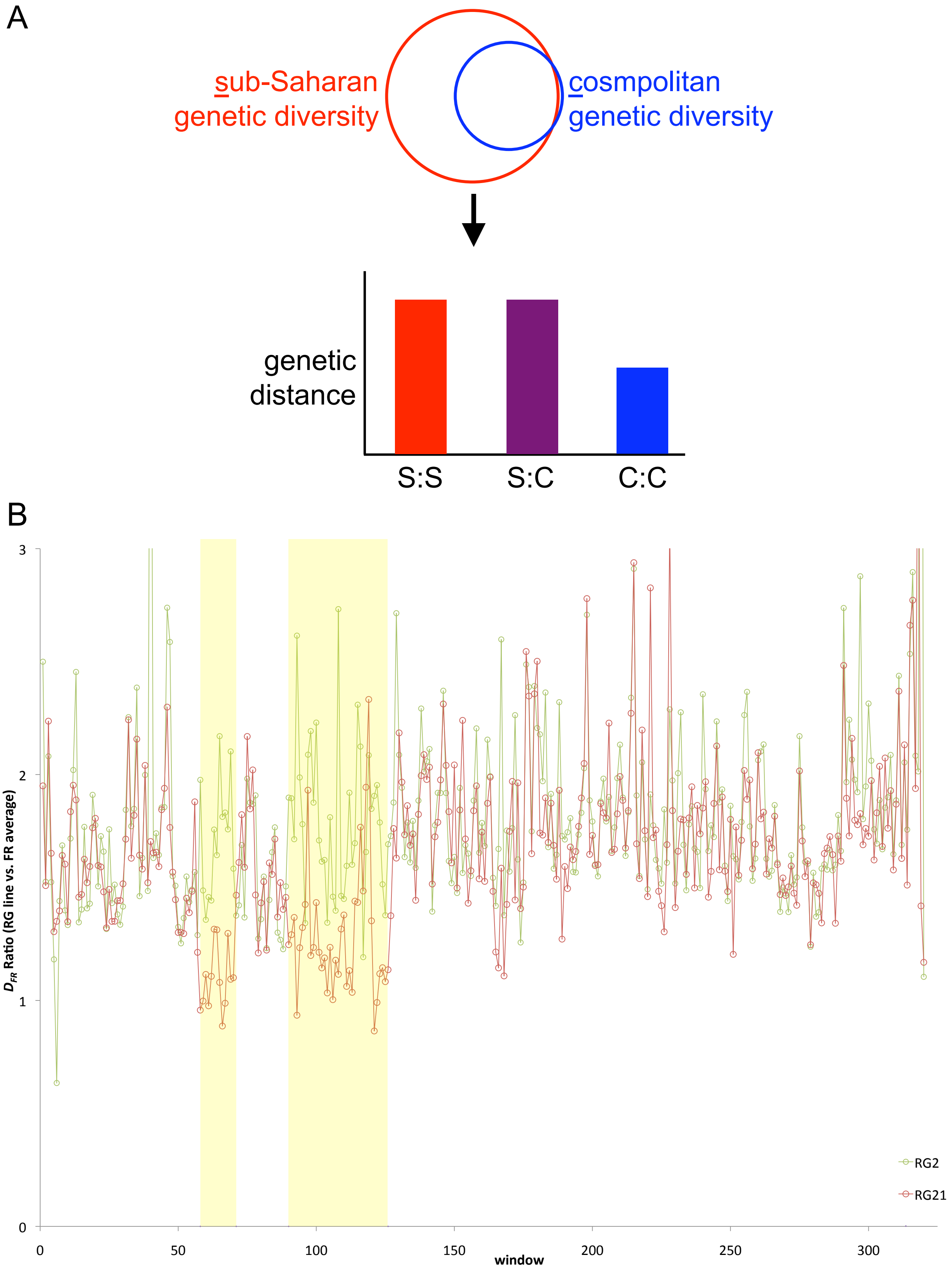
A
sub-Saharan genetic diversity
cosmpolitan genetic diversity
genetic distance
S:S
S:C
C:C
B
3
2
1
0
D_{FR} Ratio (RG line vs. FR average)
0
50
100
150
200
250
300
window
RG2
RG21

Figure S3. Expectations and observations for genetic distances with regard to population ancestry. (A) An illustration of basic diversity relationships between sub-Saharan and cosmopolitan populations. Cosmopolitan genetic variation is essentially a subset of that observed in sub-Saharan Africa. Due to the diversity loss associated with the out-of-Africa expansion, genetic distances amongst cosmopolitan haplotypes are lower than if cosmopolitan and sub-Saharan haplotypes are compared. The admixture inference method compares sub-Saharan and cosmopolitan genomes, assessing whether each genomic window truly looks like a "S:C" comparison above (in the case of African ancestry for the sub-Saharan genome) or if it instead resembles a "C:C" comparison between cosmopolitan genomes, based on genetic distance to France being lower than expected for a truly African haplotype. (B) Plots of the local ratio of D_{FR} (genetic distance to the France sample) for a single Rwanda RG genome versus the FR average (genetic distance among France genomes). Shown are RG2 in light green (for which no admixture was called) and RG21 in red (for which two admixture intervals were called, see yellow boxes), for windows along the complete arm 2R. As shown here, the two RG lines have generally similar genetic distances to the France sample, but within the putative admixture intervals, RG21 becomes more similar to the cosmopolitan genomes.
