# Supplementary material for: Population Genomics of Sub-Saharan Drosophila melanogaster: African Diversity and Non-African Admixture
Source: PLoS Genet. 2012 Dec 20;8(12):e1003080. doi: 10.1371/journal.pgen.1003080 (PMC3527209; doi:10.1371/journal.pgen.1003080)
Supplement: Figure S4 — Performance of the admixture detection HMM method on simulated data. Each chart depicts the estimated admixture probability (Y axis) for each window along the chromosome (X axis), with true admixture tracts shaded. Shown here are representative simulation results for admixture beginning 100 generations ago (A–C), 1,000 generations ago (D–F), or 10,000 generations ago (G–I). (PDF) [file pgen.1003080.s004.pdf]

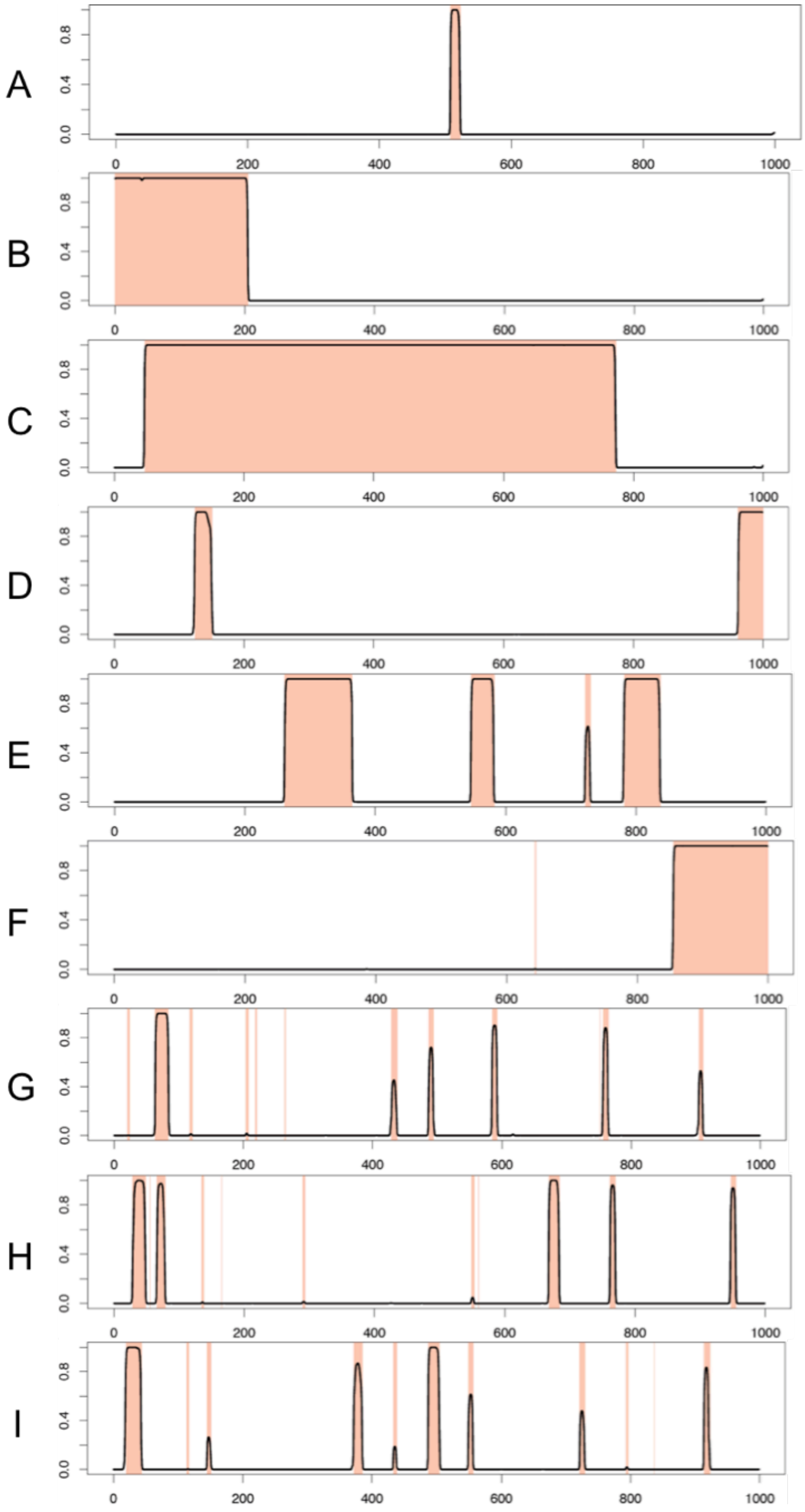
A
B
C
D
E
F
G
H
I
0.0
0.4
0.8
0
200
400
600
800
1000

Figure S4. Performance of the admixture detection HMM method on simulated data. Each chart depicts the estimated admixture probability (Y axis) for each window along the chromosome (X axis), with true admixture tracts shaded. Shown here are representative simulation results for admixture beginning 100 generations ago (A-C), 1,000 generations ago (D-F), or 10,000 generations ago (G-I).
