# Supplementary material for: Population Genomics of Sub-Saharan Drosophila melanogaster: African Diversity and Non-African Admixture
Source: PLoS Genet. 2012 Dec 20;8(12):e1003080. doi: 10.1371/journal.pgen.1003080 (PMC3527209; doi:10.1371/journal.pgen.1003080)
Supplement: Figure S5 — A log-scale plot of admixture probabilities from all genomic windows of four subsets of the sequenced African genomes. For groups of genomes within the Rwanda RG population sample and outside it, and for genomes in the primary core and secondary core categories (the former with greater than 25X sequencing depth), the proportion of window admixture probabilities within each 5% bin is plotted. The greater occurrence of intermediate admixture probabilities for secondary core genomes may indicate less accurate performance, relative to that observed for primary core genomes. (PDF) [file pgen.1003080.s005.pdf]

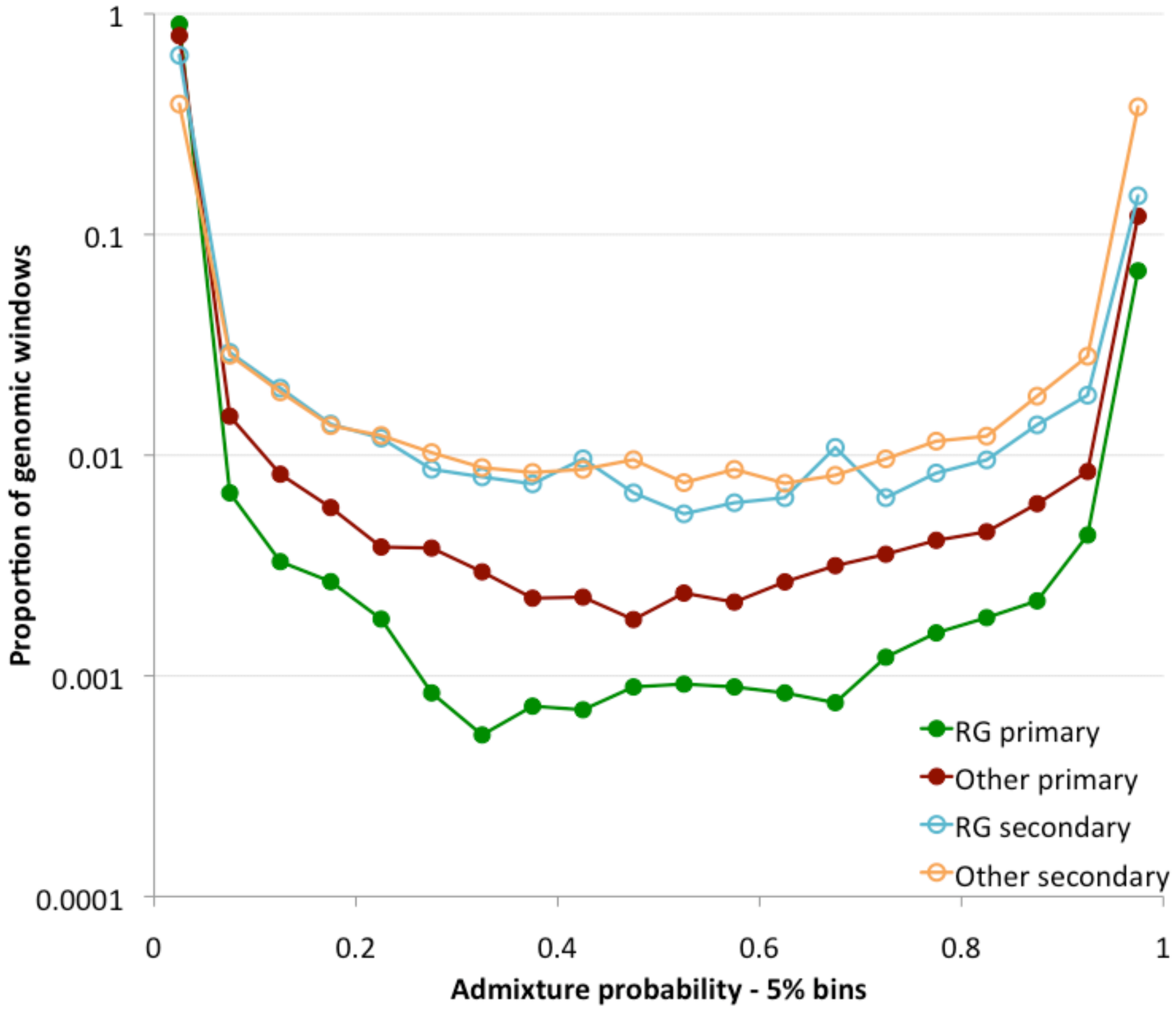

Figure S5. A log-scale plot of admixture probabilities from all genomic windows of four subsets of the sequenced African genomes. For groups of genomes within the Rwanda RG population sample and outside it, and for genomes in the primary core and secondary core categories (the former with greater than 25X sequencing depth), the proportion of window admixture probabilities within each 5% bin is plotted. The greater occurrence of intermediate admixture probabilities for secondary core genomes may indicate less accurate performance, relative to that observed for primary core genomes.
