# Supplementary material for: Population Genomics of Sub-Saharan Drosophila melanogaster: African Diversity and Non-African Admixture
Source: PLoS Genet. 2012 Dec 20;8(12):e1003080. doi: 10.1371/journal.pgen.1003080 (PMC3527209; doi:10.1371/journal.pgen.1003080)
Supplement: Figure S6 — The relationship between inferred population admixture proportion and the human population size of the collection locality. Admixture proportion is the average level of non-African ancestry estimated for a population's genomes by the HMM method described in the text. A maximum population size of 100,000 was based on the assumption that flies in larger cities continue to occupy similarly uniform urban environments. The relationship was statistically significant (Spearman ρ = 0.60; one-tailed P = 0.003). (PDF) [file pgen.1003080.s006.pdf]

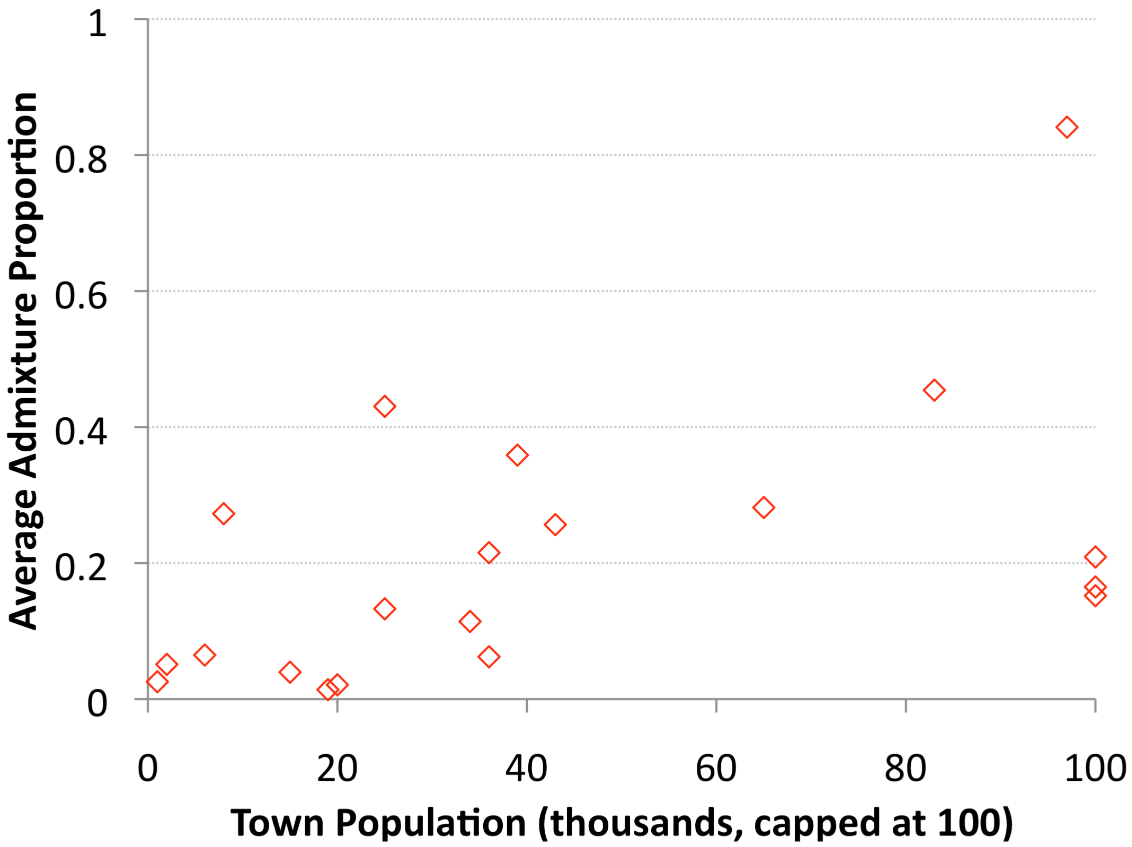

Figure S6. The relationship between inferred population admixture proportion and the human population size of the collection locality. Admixture proportion is the average level of non-African ancestry estimated for a population's genomes by the HMM method described in the text. A maximum population size of 100,000 was based on the assumption that flies in larger cities continue to occupy similarly uniform urban environments. The relationship was statistically significant (Spearman ρ = 0.60; one-tailed P = 0.003).
