# Supplementary material for: Population Genomics of Sub-Saharan Drosophila melanogaster: African Diversity and Non-African Admixture
Source: PLoS Genet. 2012 Dec 20;8(12):e1003080. doi: 10.1371/journal.pgen.1003080 (PMC3527209; doi:10.1371/journal.pgen.1003080)
Supplement: Figure S7 — Population diversity ratios across the genome. (A) France (FR) vs. Rwanda (RG) illustrates different levels of non-African diversity loss for each major chromosome. (B) FR vs. Zambia (ZI) demonstrates that results from (A) are not driven by the RG-specific patterns. (C) RG vs. ZI shows less heterogeneity, and suggests that the peak observed in (B) is due to a ZI-specific loss of diversity around the chromosome 3 centromere. Chromosome arms are labeled and indicated by color. Each window contains 5000 RG non-singleton SNPs. (PDF) [file pgen.1003080.s007.pdf]

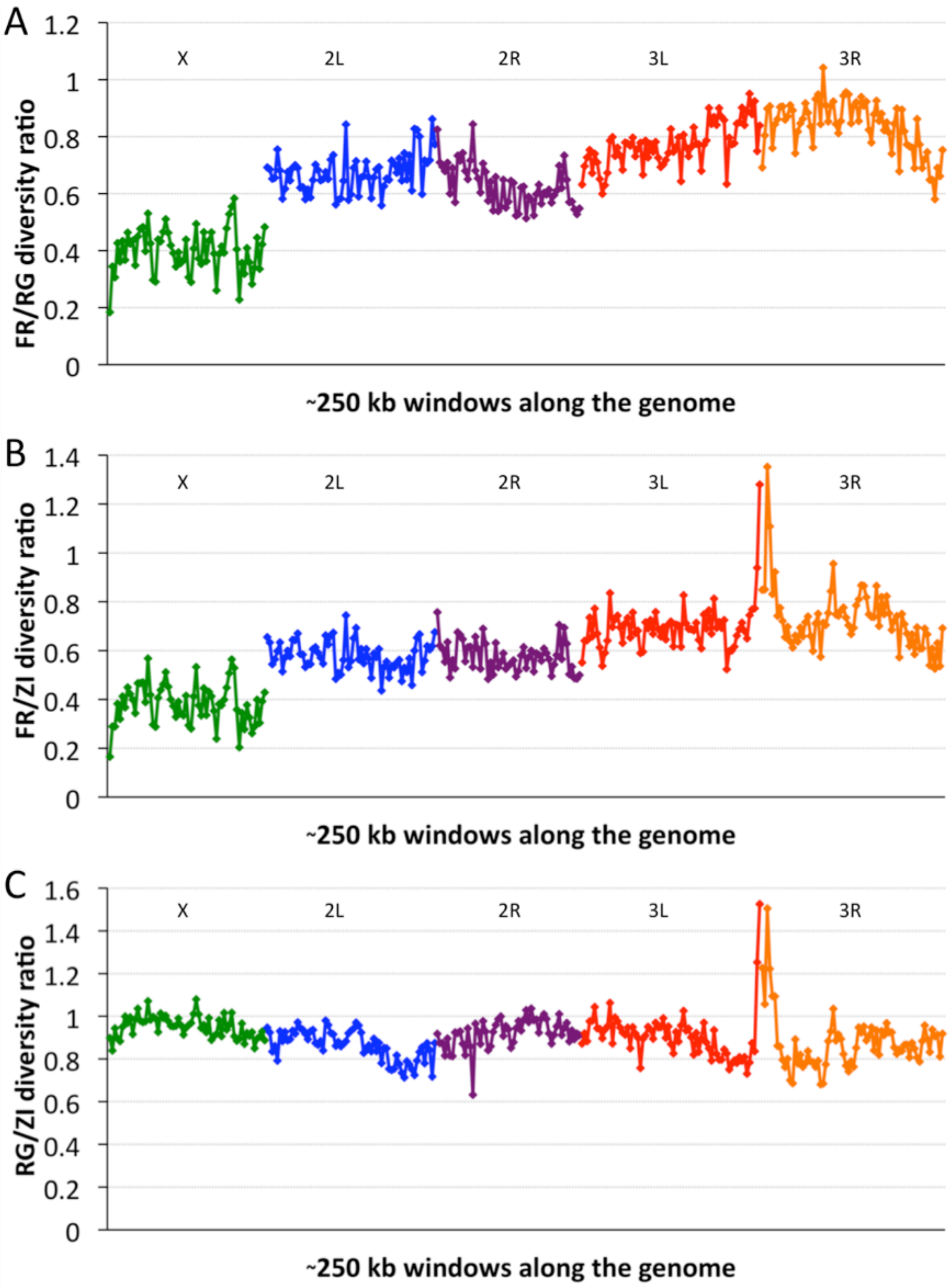
A
FR/RG diversity ratio
X
2L
2R
3L
3R
~250 kb windows along the genome
B
FR/ZI diversity ratio
X
2L
2R
3L
3R
~250 kb windows along the genome
C
RG/ZI diversity ratio
X
2L
2R
3L
3R
~250 kb windows along the genome

Figure S7. Population diversity ratios across the genome. (A) France (FR) vs. Rwanda (RG) illustrates different levels of non-African diversity loss for each major chromosome. (B) FR vs. Zambia (ZI) demonstrates that results from (A) are not driven by the RG-specific patterns. (C) RG vs. ZI shows less heterogeneity, and suggests that the peak observed in (B) is due to a ZI-specific loss of diversity around the chromosome 3 centromere. Chromosome arms are labeled and indicated by color. Each window contains 5000 RG non-singleton SNPs.
