# Supplementary material for: Population Genomics of Sub-Saharan Drosophila melanogaster: African Diversity and Non-African Admixture
Source: PLoS Genet. 2012 Dec 20;8(12):e1003080. doi: 10.1371/journal.pgen.1003080 (PMC3527209; doi:10.1371/journal.pgen.1003080)
Supplement: Figure S8 — A neighbor-joining population distance tree based on the matrix of Dxy values. Branch lengths are to scale, and basal node was obtained by midpoint rooting. (PDF) [file pgen.1003080.s008.pdf]

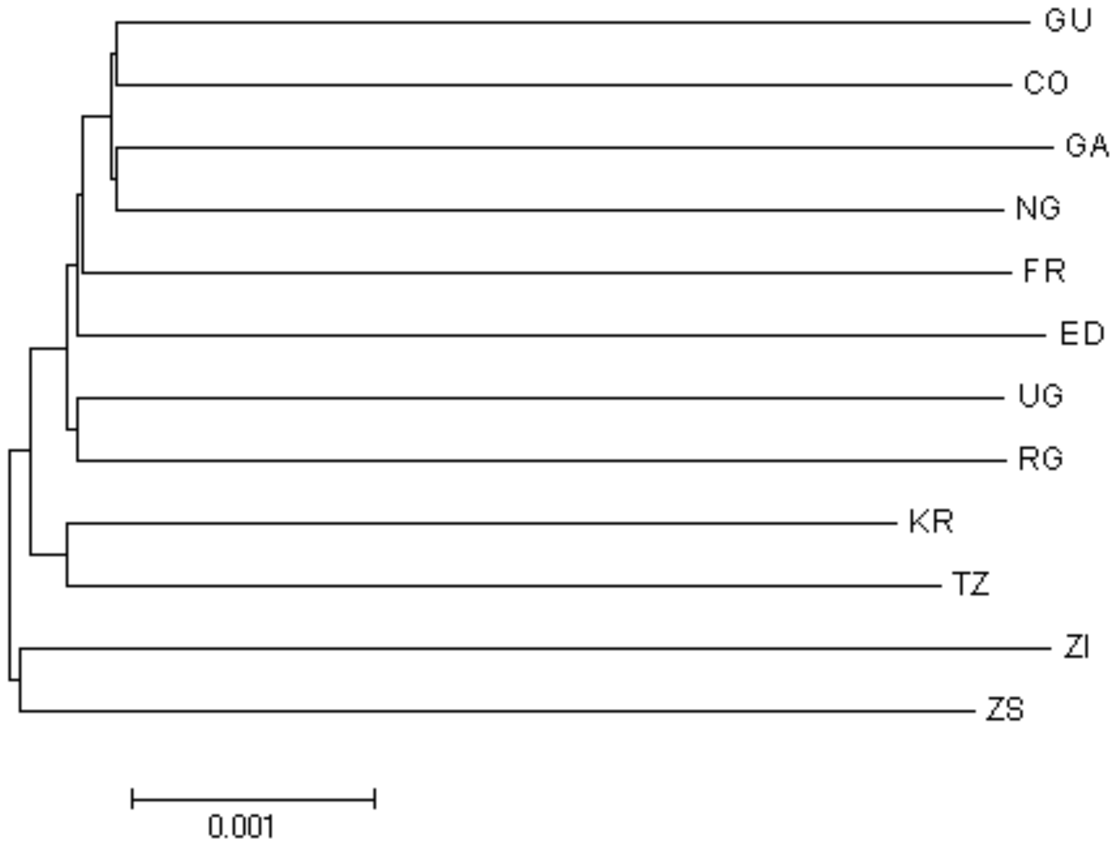

Figure S8. A neighbor-joining population distance tree based on the matrix of D_{xy} values. Branch lengths are to scale, and basal node was obtained by midpoint rooting.
